# A new measure for the attitude to mobility of Italian students and graduates: a topological data analysis approach

**DOI:** 10.1007/s10260-022-00666-x

**Published:** 2022-10-24

**Authors:** Martina Vittorietti, Ornella Giambalvo, Vincenzo Giuseppe Genova, Fabio Aiello

**Affiliations:** 1 Department of Economics, Business and Statistics, University of Palermo, Palermo, Italy; 2 Department of Economics and Law, University ’Kore’ of Enna, Enna, Italy

**Keywords:** Mobility analysis, Brain drain, Mobility index, Topological data analysis, Graph, Persistence diagram

## Abstract

Students’ and graduates’ mobility is an interesting topic of discussion especially for the Italian education system and universities. The main reasons for migration and for the so called brain drain, can be found in the socio-economic context and in the famous North–South divide. Measuring mobility and understanding its dynamic over time and space are not trivial tasks. Most of the studies in the related literature focus on the determinants of such phenomenon, in this paper, instead, combining tools coming from graph theory and Topological Data Analysis we propose a new measure for the attitude to mobility. Each mobility trajectory is represented by a graph and the importance of the features constituting the graph are evaluated over time using persistence diagrams. The attitude to mobility of the students is then ranked computing the distance between the individual persistence diagram and the theoretical persistence diagram of the stayer student. The new approach is used for evaluating the mobility of the students that in 2008 enrolled in an Italian university. The relation between attitude to mobility and the main socio-demographic variables is investigated.

## Introduction

International youth and students’ mobility is a worldwide phenomenon of interest and it has been the topic of many recent studies (Brooks and Waters 2011; Caruso and De Wit 2015; Chessa 2010; Findlay 2011; Findlay et al. 2012; Guruz 2011; Prazeres et al. 2017; Solimano 2008; Varghese 2008; Voight et al. 2020; Welsh 2017). In Europe, the main reasons of youth mobility and its interaction with the socio-economic conditions are deeply investigated by King et al. (2016). In Italy, youth and more specifically students’ and graduates’ mobility is extensively discussed by Bacci et al. (2008); Cattaneo et al. (2017); Dotti et al. (2013); Panichella (2013); Tosi et al. (2019). The interest in the Italian case derives mainly by key education statistics produced by European-Commission in 2016 that showed that Italy is one of the countries with fewest graduates compared to other European countries. The poor financial supports, the reduction of public funds for university system, the higher university fees are some of the possible causes of the decrease in the enrolment rates in the Italian universities (De Angelis et al. 2016) and of the consequent migration. Empirical researches on students’ mobility focus primarily on the micro- and macro-level determinants of mobility choices. Fewer studies, instead focus on the definition of “movers”. The most common definition, proposed in Attanasio and Enea (2019) and later employed e.g. in Columbu et al. (2021b); Genova et al. (2021), identifies as “movers” those students which i) enrol in universities that are not located in the region where their families live ii) take more than 90 min to reach the university; as “stayers” the others. Rizzi et al. (2021) propose an intermediate category “half mover” for identifying who moves in neighbouring regions. Ballarino and Panichella (2021) extend this definition including variables such as type of episode rural–urban (from rural to urban, from rural to rural, from urban to urban and from urban to rural), distance and time, but in the models they still use the classical definition of stayers and mover. This paper finds its place in this context with the aim of describing and measuring the Italian students’ mobility in time. In particular, in this paper we propose a new methodology for understanding mobility dynamic over time and space.

For giving a spatial-temporal characterisation of the phenomenon and of its entity in the different geographical areas, an innovative approach based on Topological Data Analysis is proposed. Topological Data Analysis (TDA) is a relatively new approach to data analysis that has provided quite new insights in the study of qualitative features of data (Chazal et al. 2017). It combines tools from the two disciplines of Statistics and Algebraic Topology. We can refer to TDA as the discipline that studies the “shape” of data and recognises patterns in them (Munch 2017). In the past years, applications of typical TDA tools such as persistence diagrams and summary related statistics have exponentially increased. From Materials Science (Vittorietti et al. 2020), to Neuroscience (Biscio and Møller 2019), to Biology (Kanari et al. 2018), TDA proofs its power in explaining the intricate hidden structure of the data. However, its application to social sciences is still in development (Dlotko et al. 2019). It is indeed not easy to perform a quantitative analysis or give a geometrical interpretation of something that it is intrinsically qualitative. The first step, hence, is representing the mobility data geometrically. Ignacio and Darcy (2019) show the use topo-algebraic methods to identify flow patterns in the Asian net migration and remittance networks resembling cycles involving multiple countries. In this work, the mobility trajectories of the students are represented by directed graphs made of vertices representing the locations in which the student lived and edges representing the transitions made by the student. The number of edges, or equivalently the number of connected components, and the number of loops of the graph give a measure of the ability to move of the students. However, these quantities represent the mobility in a static way, depending on the time observation considered. Furthermore, looking at the graph corresponding to the last observation time, it is not possible to distinguish the graph of a student that moved immediately after his/her diploma and the graph of a student that moved for working reason. Using persistence homology we try to give a dynamical and unique representation of the mobility trajectories of the student over time. Persistent homology is the branch of TDA that provides tools for identifying qualitative features of data and to give a measure of the importance of those features (Ghrist 2008). In the specific case, we want to study and record the evolution of features such as the number of connected components and the number of loops using persistence diagram. Persistence diagrams contain information about the lifetime and the importance of each topological features. The idea is that the more time a student spend in a specific location, the longer would be the lifetime of the topological feature associated to this event; the more transitions a student experiences the higher would be the number of points in the diagram. Comparing persistence diagrams using standard (bottleneck and Wasserstein distances) and non standard distance measures, we are able to rank the mobility of students from the grade of “stayer” to the grade of “highly mover”. The new approach is applied for studying the Italian students’ mobility. The data used for the analysis are national student level micro-data from 2008 to 2016. More precisely, they are relative to all the students that in 2008 enrolled in an Italian University longitudinally followed up to 2017.

The place of residence of the students every year is used for constructing the graphs and the new mobility index is used for investigating which are the groups of students with higher mobility. The paper structure is the following. In Sect. 2, an overview of the main reasons/causes of the Italian mobility is reported. In Sect. 3 we review the basic concepts of graph theory (Sect. 3.1) and persistence homology (Sect. 3.2) using as illustration different hypothetical students trajectories. In Sect. 3.3 distance measures between persistence diagrams are discussed and a new mobility index is proposed.

In Sect. 4 the method is applied for investigating the Italian students’ mobility. In Sect. 4.1 the data structure is described. Descriptive statistics are used to describe the main socio-economic characteristics of the Italian students and the mobility phenomenon. Then, the relation between the topological features of the graphs, such as number of vertices and loops, and socio-economic characteristics of the students such as, gender, disciplinary area of study, geographical area of origin, degree of study is investigated in Sect. 4.2. Afterwards, the persistence diagrams are computed. The new mobility index, based on the scaled distance between the persistence diagram of the student under analysis and the archetype persistence diagram of the stayer is calculated. The new variable is studied in relation with the socio-economic characteristics of the students. Interpretation of the results, remarks and final considerations are discussed in Sect. 5.

## Theoretical framework

Mobility is a multi-dimensional phenomenon that has been investigated over the years using both macro-determinants, such as the social context, the labour market, and micro-determinants, such individual choices and personal skills. At a macro-level, factors such as unemployment and education are the most common listed causes of mobility. More in general, the root of mobility and migration can be found in the economic context. For instance, during the severe worldwide financial crisis in 2007–2008 (the global financial crisis, GFC), a significant increase in mobility has been registered (Staniscia et al. 2019). The scale and the time of the recession varied from country to country hitting mainly North America, South America and Europe. The effects of the GFC were visible looking at the trend of the main economic indicators: the annual real world GDP per capita experienced a strong decline; the unemployment rate (especially the young unemployment rate) grew significantly; household incomes and funds for public services contracted. The relation among these indicators and youth mobility has been the focus of several studies. In particular, youth unemployment due to the post-2008 economic crisis is listed as one of the main factors influencing mobility (King et al. 2016). There exists a strong connection between unemployment and mobility: this is the main conclusion of Allegro and Giambalvo (2020); Barrioluengo and Flisi (2017); Di Pietro et al. (2005); Iammarino and Marinelli (2015); Losurdo et al. (2013); Nifo and Vecchione (2014); Tintori and Romei (2017); Türk (2019); Vittorietti et al. (2019). Bacci et al. (2008); Cattaneo et al. (2017); Ciriaci (2014); Dotti et al. (2013); Panichella (2013); Tosi et al. (2019) find a correlation between the flows of mobility for study and those for work purposes. In particular, Iammarino and Marinelli (2015) face the problem of education-job (mis)match and correlate it to the interregional migration finding out a positive role of the interregional mobility on increasing the probability of finding a job.

In this scenario, the educational system becomes crucial in creating human capital for the economies. In fact, education plays a fundamental role in the development of work achievements, especially in the occupational status and the income (Card 1999; Schneider 2010). Growth rates of economies are determined by the performance of the knowledge-based sectors (Chen and Dahlman 2004). Knowledge has become a good, and the institutions that produce knowledge (like universities) influence countries development (Varghese 2008). The Europe strategy ET 2020 (strategic context for European cooperation in education field), establishes that the value of graduates employment rate and more generally the youth employment rate (20–34 years old) in 2020 should be 82% (Stumbriene et al. 2020).

Furthermore, recent studies show that a higher qualification is necessary for achieving healthier lives, for increasing the probability of being appointed with better salary and for a more gratifying job (Powdthavee et al. 2015; Schuck and Steiber 2018; Zambon 2019). Therefore, universities are responsible for offering services that can contribute to higher opportunities for students and graduates. On the one hand, universities are subject to yearly evaluation and ranking based on offered services, research outcomes, placement results and so on: this increases competition among the universities ( Bowman and Bastedo 2009) and it influences student applications, enrolment decisions and also students’ mobility (Biancardi and Bratti 2019). From recent empirical studies, it emerges that the specific quality and the location of the colleges, besides affecting students’ and graduates’ mobility (Giambona et al. 2017), have an influence also on their employment probabilities (Brunello and Cappellari 2008) and returns (Makovec 2006).

In Italy, the attractiveness of an university is not only associated with the quality of the services offered and its prestige but also with the characteristics of the labour market in which the university operates (Dal Bianco et al. 2010; Dotti et al. 2013; Grossi and Serra 2002). In some cases, students’ mobility is really about the institutional characteristics more than territorial ones. For instance, the specific University research performance is very relevant for PhD mobility (Barrioluengo and Flisi 2017).

On the other hand, the outgoing mobility of highly-skilled people, commonly referred as “brain drain” (Milio et al. 2012), is itself one of the criteria used for ranking universities and hence universities with low ranking position experience difficulties in improving their condition.

However, mobility can also be seen as an opportunity. Nowadays, there are several initiatives and reforms, starting from the Bologna Process, such as the Erasmus project, created with the aim of enhancing the quality and recognition of European higher education systems and of improving the conditions for exchange and collaboration within Europe. Both the Bologna Process and the general globalisation trend seem to support students’ mobility in some aspects (Rumberger and Larson 1998). Students’ mobility can have a positive connotation: it can be perceived as a relatively well-documented growth trend of a phenomenon mostly viewed a highly desirable (King et al. 2010).

The moving can be vertical, towards economically more advanced and academically superior systems; or horizontal, that is, between countries or institutions of more or less equal level of academic quality.

The vertical mobility in Italy has a double meaning: it is a geographical vertical mobility, observed from the southern regions to the northern ones and a social vertical mobility, in terms of improvement of employment and lifestyle. Studies on Italian domestic students’ mobility (Attanasio and Enea 2019; Enea 2016; Giambona et al. 2017) and graduates’ mobility (Iammarino and Marinelli 2015; Panichella 2013), confirm that the “Mezzogiorno” is experiencing a proper brain drain to the Centre-North of Italy. The regions of South-Italy are characterised by a very low employment rate. The difference between South and North is around 30 percentage points: namely 44.8% for the South versus 67.9% for North (Istat, 2019). A similar tendency is observed for the graduates’ unemployment rate in 2019: in Southern regions 24.9% versus 11.9% in Northern regions. The report about Families and Labour Market (Istat, 2019) highlights recovery from pre-crisis levels of unemployment in all Northern regions but not in all the Southern regions.

The territorial inequalities in Italy, strictly related to the well-known North–South divide, are mirrored by the different attitude and different performance of students and graduates of Northern and Southern universities. This results in different students’ mobility trajectories. The geographical gap between North and South accentuates the brain drain phenomenon in the southern regions (Res and Viesti 2016). Southern students are forced to migrate to the central and northern regions of the country because of the lack of job in Southern regions. This is confirmed by a significant decrease of students enrolment in Southern universities in favour of an increase in northern and central universities (Attanasio and Enea 2019; Res and Viesti 2016). This tendency creates further inequalities within the country as well as a cultural and socio-economic losses for the South. So, this substantial brain-drain in the South is a selective process that represents an obstacle to the economic and cultural development in the southern areas.

In this case, students’ mobility is not a choice. At a micro-level, young people and students migration is influenced by personal experiences, by home obligations and in general by the macro-context where they are situated (Van Mol 2014; MacDonald and Marsh 2005; Serracant 2012; Farrugia and Wood 2017). Students’ mobility is the result of individual decisions reflecting personal characteristics, for example, socio-economic and personal skills (Zambon 2019; Findlay et al. 2012; Dreher and Poutvaara 2011; King 2002, 2009). Students are usually inclined to move and change their place of residence in order to increase their education and to find a job (Card 1999; Schneider 2010). The socio-economic status, the labour market, the educational paths, the personal choices, the individual skills and the intricate relations among them makes the identification of the structure and of the dynamic of mobility very challenging.

## Methodology

The aim of this section is to represent the mobility trajectories with graphs and to study the features of a special structure describing it over time using persistence homology. The relation between graph theory and persistence homology is extensively investigated by Bergomi et al. (2017); Horak et al. (2009); Otter et al. (2017). In this paper, first we briefly and intuitively introduce the necessary notations and definitions of graphs (see Livi and Rizzi 2013) for more formal definitions), second we give a built-in explanation of the persistence homology approach through an example based on hypothetical Italian mobility data, finally we introduce the new mobility index based on persistence diagrams differences.

### Graph representation

A graph, *G*, is usually defined as a structure or diagram made of points also called vertices or nodes *V*(*G*), and lines connecting the points called edges *E*(*G*).

We consider *directed* graph or graph in which the edge that connects two vertices (*A*, *B*) is not the same of the one that connects (*B*, *A*). An edge is considered *mutual* if in a direct graph both the edge that connects (*A*, *B*) and the edge that connects (*B*, *A*) exists. A *loop* in a graph is an edge that connects a vertex to itself. A graph is called a *dynamic* (time-varying) graph if the graph varies over time, i.e. it might present vertex and edge deletions and additions (Aktas et al. 2019).

A graph in this paper represents the mobility trajectories of the Italian students. The graph vertices represent the locations visited by the students. The edges represent the transitions. The loops represent the permanence for more than one observational time in the same location. A theoretical example of hypothetical Italian internal mobility trajectories (studied in more detail in Sect. 4) can clarify the meaning of the graph features in the case under study. The code list of the Italian provinces used in the example is provided in Table  5 in the Appendix.

**Fig. 1:**
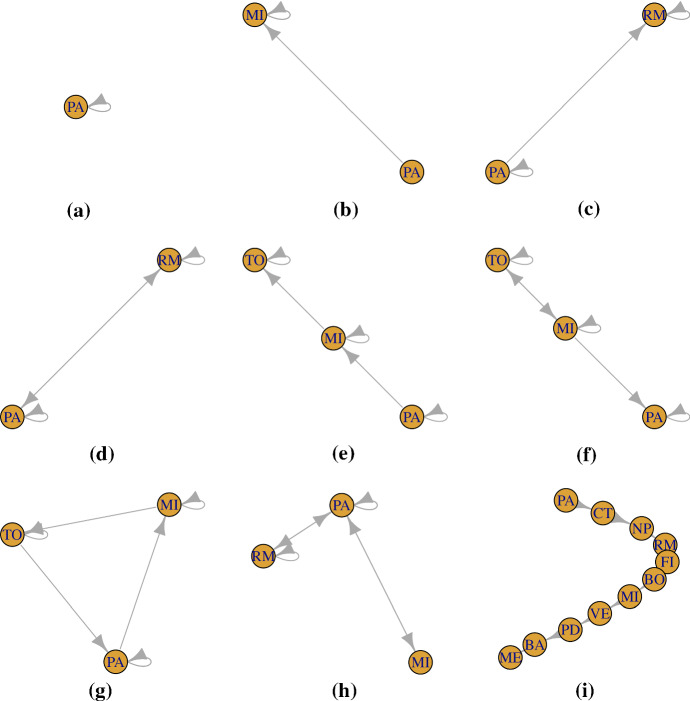
Graph representation of the mobility trajectories of nine hypothetical students: the graph vertices represent the locations visited by the students; the edges represent the transitions; the loops represent the permanence for more than one observational time in the same location

Let us consider the hypothetical mobility trajectories of 9 students (Fig. 1):

- The first student gets his diploma in Palermo; he enrols at the University of Palermo in 2008 and he obtains his Bachelor’s Degree (BSc) in 2011. He completes his University career with a Master’s Degree (MSc) in 2013. He still lives in Palermo.
- The second student gets her diploma in Palermo; she enrols at the University of Milan in 2008 and there, she gets her BSc in 2011. In the same year she enrols in a Master’s Degree course at the same University and she graduates in 2013. She immediately finds a job in Milan and she keeps living there.
- The third student gets her diploma in Palermo; she enrols at the University of Palermo in 2008 and she obtains her MSc in 2013. Afterwards, she decides to enrol to a specialistic course in Rome. She gets her specialisation and she still lives in Rome.
- The fourth student gets her diploma in Palermo but she enrols at the University of Rome in 2008 and she obtains her BSc in 2013. Afterwards, she comes back to Palermo.
- The fifth student gets his diploma in Palermo and he enrols at the University of Palermo in 2008. However, he would like to study in Milan, therefore after one year he moves there. He obtains his BSc in 2012 and he enrols in a Master’s course in Turin. He completes his University career with a MSc in 2015. He still lives in Turin.
- The sixth student presents a case very similar to student (e) case but he, after his Master’s Degree, finds a job in Milan and moves back there.
- The seventh student presents also a similar case to the one of students (e, f), but he after his Master’s goes back to Palermo.
- The eighth student gets her diploma in Palermo but she enrols at the University of Milan in 2008. After one year she goes back to Palermo. She enrols at University of Palermo and she obtains her Bachelor’s Degree at University of Palermo. Afterwards, she enrols in a Master’s Degree course in Rome, but again she goes back to Palermo after her Master’s Degree graduation.
- The last student gets his diploma in Palermo and afterwards changes his location every year, looking for the right place to settle.

So, for a fixed time *t* we can compare and evaluate the graphs in terms of the geometrical features previously defined. Note that other important graph features such as cliques and triangles should be taken into account to compare graphs; here we just show the features that will be used in the subsequent analysis. In Table 1, the number of vertices ($n_{v}$), of mutual edges ($n_{\bar{e}}$) and of loops ($n_{l}$) of the graphs of the 9 students previously described, are compared. Note that the number of edges is equal to the number of vertices -1, therefore just one of them is reported.

**Table 1:** Main geometrical characteristics of the graphs of the nine hypothetical students: number of vertices ($n_{v}$), number of mutual edges ($n_{\bar{e}}$) and number of loops ($n_{l}$)

Student	$n_{v}$	$n_{\bar{e}}$	$n_l$
(a)	1	0	1
(b)	2	0	1
(c)	2	0	2
(d)	2	1	2
(e)	3	0	3
(f)	3	1	4
(g)	3	0	4
(h)	3	2	3
(i)	11	0	1

It is worth to notice that the graph representing the mobility trajectory of student (a) (Fig. 1a) is the most basic graph or the graph of the *stayer* student. Therefore, we define the *stayer graph* or *minimum graph* as the graph made of one component and one loop. The graph representing the mobility trajectory of student (i) (Fig. 1i) represents the highest observed mobility. Therefore, we define the *highly mover graph* or the *maximum graph* as the graph made of the maximum number of components and no loops. Graph comparison is an important task for many graph applications such as classification and matching. The most common ways proposed in the literature are graph isomorphism (exact matches) (Cordella et al. 2004) graph edit distance or some measures of structural similarity (Gao et al. 2010), graph kernels (Shervashidze et al. 2011). Most of the proposed methods relies on the comparison between graph geometrical features considering the configuration of the graph at a specific time observation. In recent years, persistent homology has been proposed as a useful tool to extract and compare topological features of graphs and networks. Persistent homology allows to take into account the dynamic structure of the graph. In the next Sect. 3.2 we introduce persistence homology and we show the advantages of using it for comparing mobility attitudes.

### Persistence homology

Topological Data Analysis (TDA) is a relatively new discipline that has provided new insights into the study of qualitative features of data (Fasy et al. 2014). In particular, persistent homology is the branch of TDA that provides tools both to identify qualitative features of data and to give a measure of the importance of those features (Chazal and Michel 2021). The main aim of persistent homology is to record the evolution of those characteristics with respect to a scale parameter *t* often representing time. The input of the analysis typically takes the form of a point cloud ${\mathcal {X}}$, an image, a geometrical structure or a graph. Based on that, a special structure, called *simplicial complex* is built. It provides information about key topological features such as connected components, loops and holes. This structure is based on the so called simplices. A geometric *k*-simplex is the convex hull of $k+1$ affinely independent points $v_0,v_1,\ldots ,v_k$. More precisely the 0-simplex identifies vertices, the 1-simplex line segments and the 2-simplex triangles. The main idea behind persistence homology is evaluating the change in the complex (when a simplex is added or removed) through a *filtering function*.

A *filtration* is a sequence of subcomplexes, $K_i$, constructed during a process, such that $\emptyset =K_0\subset K_1\subset \cdots \subset K_n=K$.

Special simplicial complexes can be constructed for directed and undirected graph and networks. Aktas et al. (2019) report the main filtrations associated to simplicial complexes representing network structures. Among them, the most commonly used are the *neighborhood complex* and *clique complex* (Jonsson 2007). The neighbourhood complex *N*(*G*) is constructed from the graph G, with vertices $V(G)=\{v_1, \ldots , v_n\}$ in such a way that for each vertex $v\in V(G)$ there is a simplex containing the vertex *v*, along with all vertices $w\in V(G)$ connected to it, and the corresponding faces. The neighbourhood complex is obtained by including all faces of those simplices.

In a graph, a clique is a set of nodes that are pairwise connected, and a maximal clique is a clique that cannot be made any larger. The vertices of the clique complex *C*(*G*) are the same as the vertices of G with the maximal complete subgraphs (cliques) as simplices so that it is essentially the complete subgraph complex. These two methods are not the only ones used for constructing simplicial complexes from graphs. Actually, any property of the graph *G* that is preserved under deletion of vertices or edges may be used for simplicial complexes’ construction. For taking into account the *dynamic* structure of the graph, filtration such as the *temporal filtration* (Pal et al. 2017) and the *zigzag simplicial filtration* (Carlsson and De Silva 2010) are more suitable. While the temporal filtration only considers the vertex and edge insertion into a dynamic graph which yields adding simplices to the simplicial complexes, the zigzag simplicial filtration allows vertex and edge deletion from a dynamic graph which yields removing simplices from the simplicial complexes. This filtration generalises standard filtrations by allowing the simplicial complexes to become smaller. Two extra conditions are needed for the zigzag simplicial filtration on a graph *G*: (i) each point in the set has a neighbourhood that includes only finitely many of the points in the set and (ii) for any scale parameter value $t\in {\mathbb {R}}$, it holds that $K_{t-\epsilon } \supseteq K_{t}\subseteq K_{t+\epsilon }$ for all sufficiently small $\epsilon >0$.

The mobility trajectories of the students can be described by an ordered sequence of subcomplexes, $K_i$ in which the 0-simplices identifies vertices and the 1-simplices the loops. Notice that in principle we could consider higher-order simplices corresponding to topological features such as triangles, however the first two homological features proved to be sufficiently discriminant in several applications (see Table 2 Aktas et al. (2019)).

At every time observation one or more simplex can appear or disappear, “born” or “die”. The idea behind persistence homology is studying the “lifetime” of the geometrical features of the structure under analysis. The “birth” and the “death” of the vertices and of the loops are plotted on an x-y plane resulting in the so called *persistence diagram*. Since the topological features can only die ‘after’ they are born ($d\ge b$), necessarily each point appears on or above the diagonal line $y = x$.

**Fig. 2:**
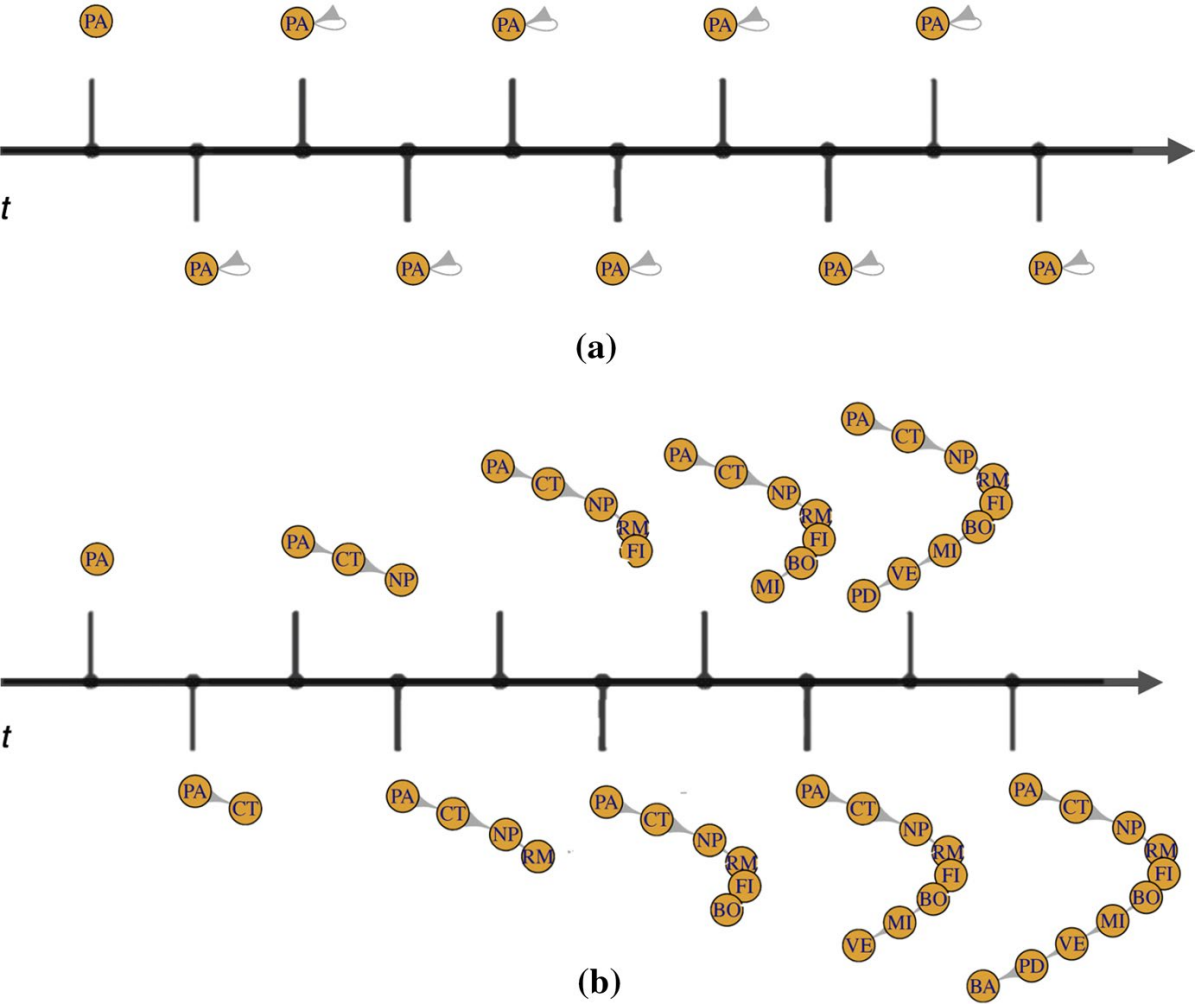
Zig-zag filtration of the graphs for the “stayer” student (a) (upper panel **a**) and for the “highly mover” student (i) (lower panel **b**)

An example can clarify the concept. In Fig. 2 the filtrations for the “stayer” and for the “highly mover” student described in Sect. 3.1 are shown.

Let us consider the filtration of the “stayer” student (a) (Fig. 2a). At time $t_0$, the year of the diploma ($<2008$) the first vertex, corresponding to the location of Palermo, appears. He enrols at the University of Palermo in 2008 and this is represented by the appearance of the loop. Student (a) spends all his career in Palermo and he never performs a transition.

**Fig. 3:**
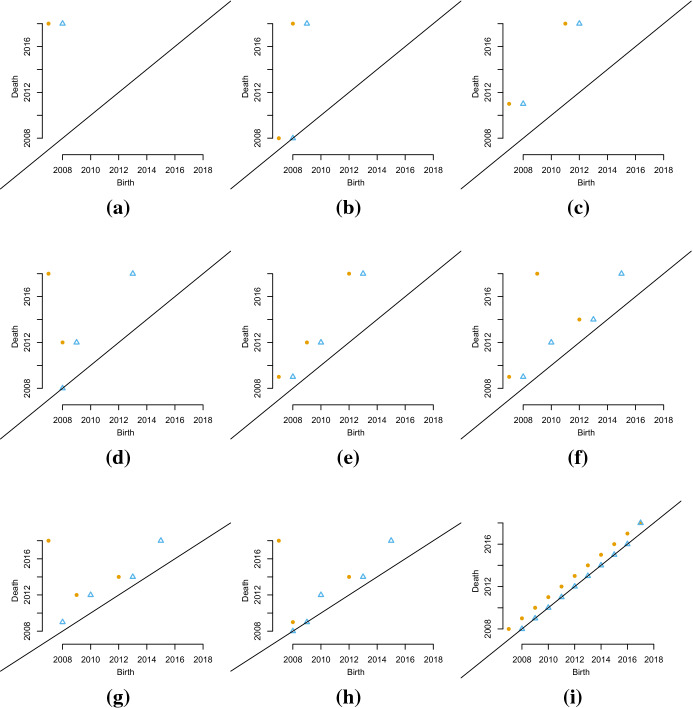
Persistence diagrams of the graphs of the nine hypothetical students. Yellow dots represent the birth- and death times of the vertices; blue triangles represent the birth- and death times of the loops (colour figure online)

The filtration corresponding to the “highly mover student” student (i) (Fig. 2b) is more eventful. At time $t_0$, the year of the diploma ($<2008$) the first vertex, corresponding to the location of Palermo, appears. Time goes and at each time observation one vertex appears, until the final graph configuration is reached. The persistence diagrams associated to the graphs of the hypothetical students are shown in Fig. 3a–i).

The dots, $D_{i}^j$ ($j=0$), represent the birth- and death times of the vertices; the triangles, $D_{i}^j$ ($j=1$), represent the birth- and death times of the loops (Fig. 3). From a graphical inspection of the persistence diagrams, it possible to notice that:

- the closest the points or the triangles are to the bisector, the least “significant” the topological feature associated to them is. In fact, a short lifetime is associated to the points/triangles that are closer to the bisector;
- the persistence diagram associated to the graph of the “stayer” student (a) is the *stayer persistence diagram* and it has just one point associated to one connected component and one triangle associated with one loop. Both have the longest lifetime;
- the persistence diagram associated to the graph of the “highly mover” student (i) is the *mover persistence diagram* and it has one point associated to each time observations; loops do not have time to form, hence their birth time coincides with their death time;
- if the points or triangles show an increasing linear trend, the mobility of the student is an outgoing mobility in which the student keeps moving and does not perform any return to previous location.

### Construction of the mobility index

In this subsection we propose a new mobility index based on the weighted Wasserstein distance between persistence diagrams. Defining a distance metric for persistence diagrams is not a trivial task and some assumptions are required (Edelsbrunner and Harer 2010; Mileyko et al. 2011; Turner and Spreemann 2020). In fact, we need to define the space of persistence diagrams and establish a *stable* metric on this space (Aktas et al. 2019). The two most common metrics used to measure similarity and distances between persistence diagrams are the bottleneck and the Wasserstein distances.

The idea behind both the two metrics is matching points of one diagram with points of another diagram, allowing the match to be done with the diagonal if necessary.

Let ${\mathcal {D}}$ denote the set of all persistence diagrams. We will consider a family of metrics which are analogous to the *p*-Wasserstein distances on the set of probability measures, and to the $L_p$ distances on the set of functions on a discrete set (Skraba and Turner 2020).

**Fig. 4:**
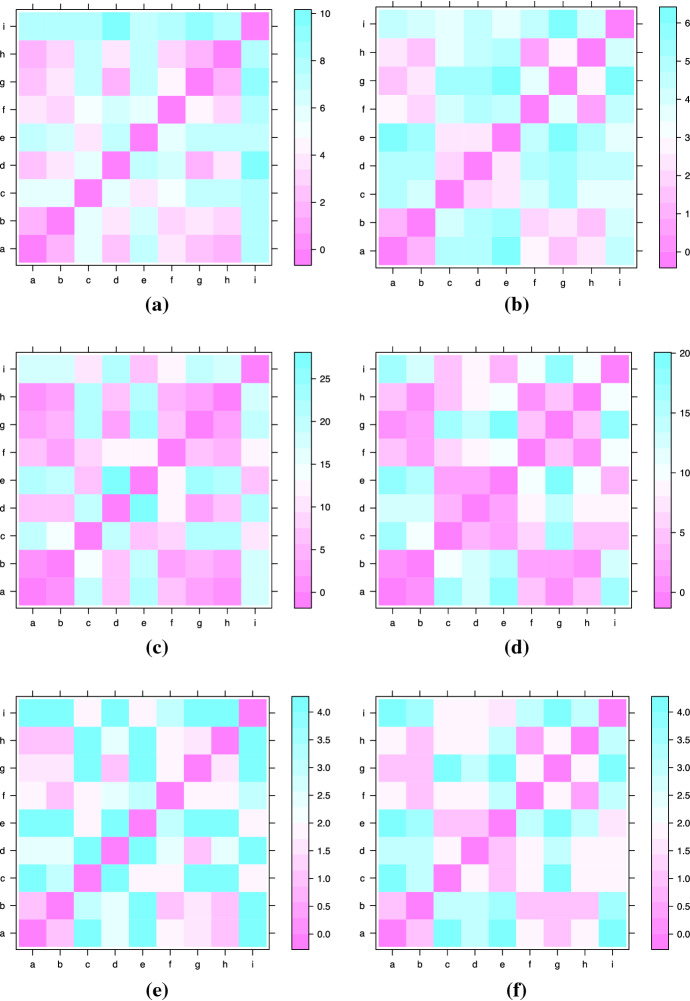
Distance matrices among persistence diagrams of the nine hypothetical students. Wasserstein Distance $p=1$ (**a**)-(**b**), Wasserstein Distance $p=2$ (**c**)-(**d**), bottleneck Distance $p=\infty$ (**e**)-(**f**) (vertices left column, loops right column)

The *p*-Wasserstein distance between two persistence diagrams, $d_1$ and $d_2$, is defined as

1 $$\begin{aligned} W_p^j(d_1,d_2)=\left( \inf _\gamma \sum _{x\in d_1}|x-\gamma (x)|^p_\infty \right) ^{\frac{1}{p}} \end{aligned}$$

where $\gamma$ ranges over all bijections from $d_1$ to $d_2$ and *j* indicates the considered dimension ($j=0$ vertices, $j=1$ loops). The set of bijections is nonempty because of the diagonal. For $p=\infty$ the resulting distance is the so called bottleneck distance. Bottleneck distance is the maximum distance between any pair of points, and thus it gives a measure for the most work that must be done to push one diagram into the configuration of the other. Wasserstein distance sums powers of the distances between the pairs; unlike the bottleneck distance, it takes all of the points into account (Munch 2017). Unfortunately, with this definition of the distance, this space is not complete, hence not appropriate for statistical inference (Fasy et al. 2014). Therefore, persistence diagrams with only finitely many off-diagonal points are considered. A graphical representation of the Wasserstein distance ($p=1$ and $p=2$) and the bottleneck distance ($p=\infty$) are shown in Fig. 4.

The three different distance metrics highlight some interesting similarities among the students:

- considering the persistence of the vertices (Fig. 4a–e) the persistence diagram of students (b) and (i) followed by the one of students (g) and (d) are the most similar to the persistence diagram of student (a) or the stayer persistence diagram. The persistence diagram of students (c), (e) and (i) instead differ notably from the one of student (a). Student (f) has an intermediate position;
- considering the persistence of the loops (Fig. 4b–f)) the persistence diagram of student (b) is the only one similar to the stayer persistence diagram.

In terms of mobility, this means that the mobility of student (d), (g) and (h) characterised by a return to their original location are similar to the one of student (a) that never moves and to the one of student (b) that moves immediately after the diploma. Students (c), (e), (f) and (i) instead have a more outgoing mobility given that they do not come back to their diploma location. Choosing which metric you wish to use to compare a set of persistence diagrams will be application specific. For larger *p* you emphasise a few (or just one in the case of $p=1$) larger differences compared to lots of smaller differences.

The aim of the paper is to give a measure of the attitude to mobility. Therefore, we propose a new index based on the distance between the observed persistence diagram and the stayer persistence diagram. Because each topological feature, vertices and loops, matters for identifying the mobility structure and dynamic, we first calculated distances for each dimension and then we combined them as a root-mean-square for the overall distance $d_i$ between the persistence diagrams *i* and the stayer persistence diagram $d_{stayer}$:

2 $$\begin{aligned} m_i=\sqrt{\alpha W_p^{j=0}(d_i,d_{stayer})+(1-\alpha )W_p^{j=1}(d_i,d_{stayer})}, \end{aligned}$$

This index is inspired by the distance measure proposed by Lee et al. (2018). In Table 2 the results for three different *p*-parameter of the Wasserstein distance are shown.

**Table 2:** Rank of the mobility attitude of the students based on the mobility index built using different *p*-Wasserstein distances

Student	$W_{p=\infty }$	$W_{p=1}$	$W_{p=2}$
(a)	0.00	0.00	0.00
(b)	1.00	1.27	1.13
(c)	4.00	5.76	19.15
(d)	3.81	4.81	19.48
(e)	5.00	7.02	27.27
(f)	3.81	6.32	20.59
(g)	3.69	5.93	20.28
(h)	3.61	5.41	20.00
(i)	5.00	8.07	26.40

Wasserstein distance with $p=1$ seems to better discriminate among the persistence diagrams of the students, showing the highest values in correspondence of the difference between the stayer persistence diagram (the persistence diagram of student (a)) and the highly mover persistence diagram (the persistence diagram of student (i)).

## The Italian case

Italy with its geographical gap, the common North–South divide, and its experience of brain drain constitutes an interesting case in mobility analysis. In the following subsections after having explained the data under analysis, the relation between the mobility index proposed in Sect. 3.3 and the principal socio-economic variables is explored.

### Descriptive analysis

For studying internal mobility, aggregate cross-sectional data coming from available sources such as Ministero dell’Istruzione e della Ricerca (MIUR), Anagrafe Nazionale Studenti (ANS), and/or from internal archive of specific universities are commonly used. The lack of longitudinal follow-up and the matching of different sources are problems that often occur in the analysis of mobility data (Ruspini 2002). The structure of these data instead allows the follow-up of the Italian students. In this paper, the focus is on Italian students that enrolled in an Italian University in 2008 longitudinally followed up until 2017 (243488 records in total). The available information are classified in three different sections: personal, high school and university data.

**Fig. 5:**
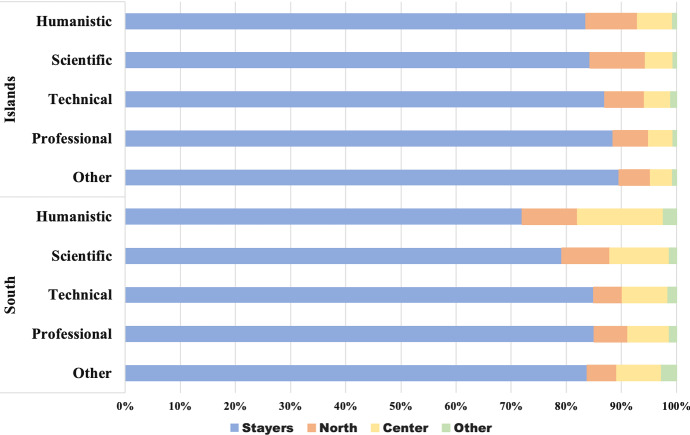
Distribution of the Italian students by high school mobility status, high school diploma and macro-region of residence

**Fig. 6:**
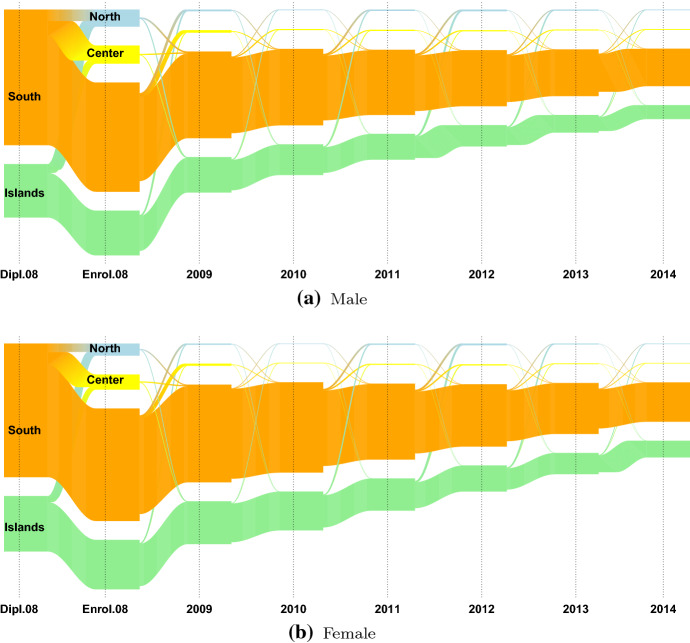
Mobility flows of the 2008 cohort of students conditioned on the macro-region of residence observed from 2008 to 2014 and the gender

**Fig. 7:**
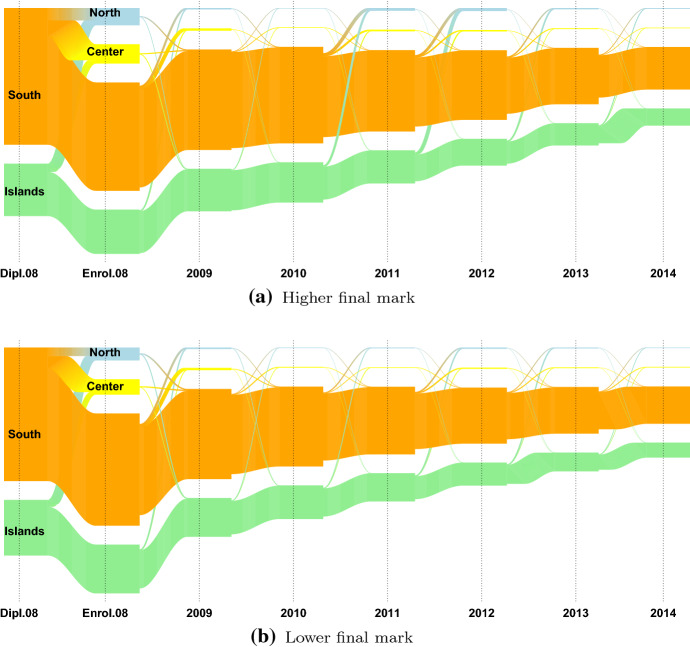
Mobility flows of the 2008 cohort of students conditioned on the macro-region of residence observed from 2008 to 2014 and the final high school mark

**Fig. 8:**
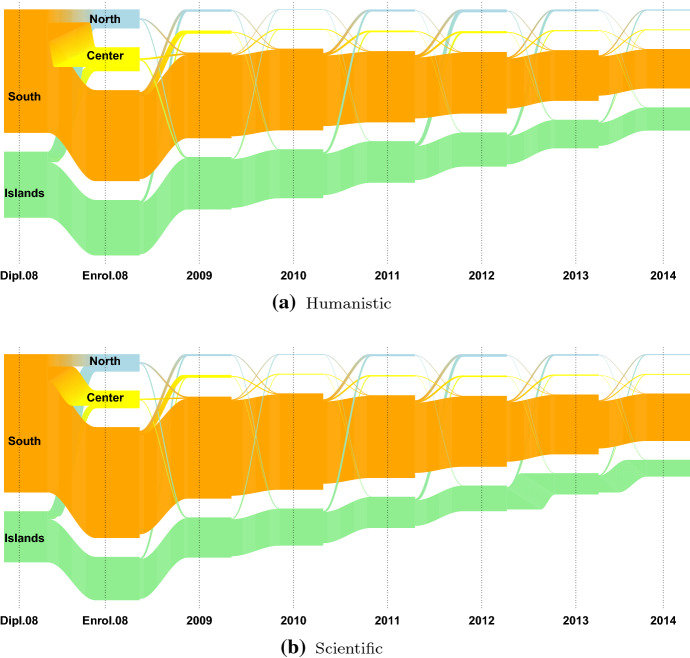
Mobility flows of the 2008 cohort of students conditioned on the macro-region of residence observed from 2008 to 2014 and the type of high school diploma

**Fig. 9:**
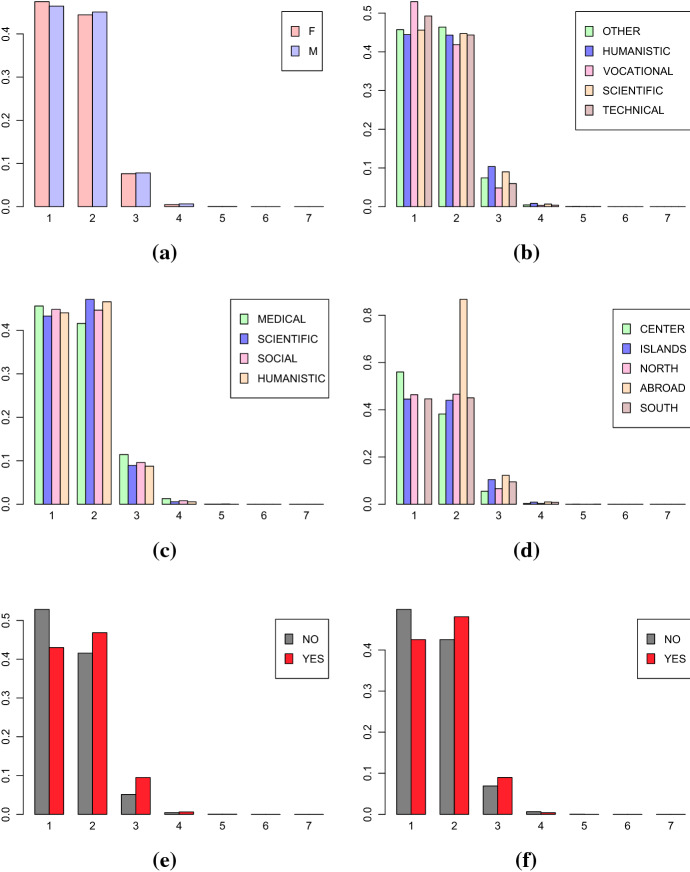
Distribution of the number of vertices in the graphs representing the mobility trajectories of every students conditioned to **a** gender, **b** high school, **c** disciplinary area, **d** geographical area, **e** Bachelor’s Degree, **f** Master’s Degree

**Fig. 10:**
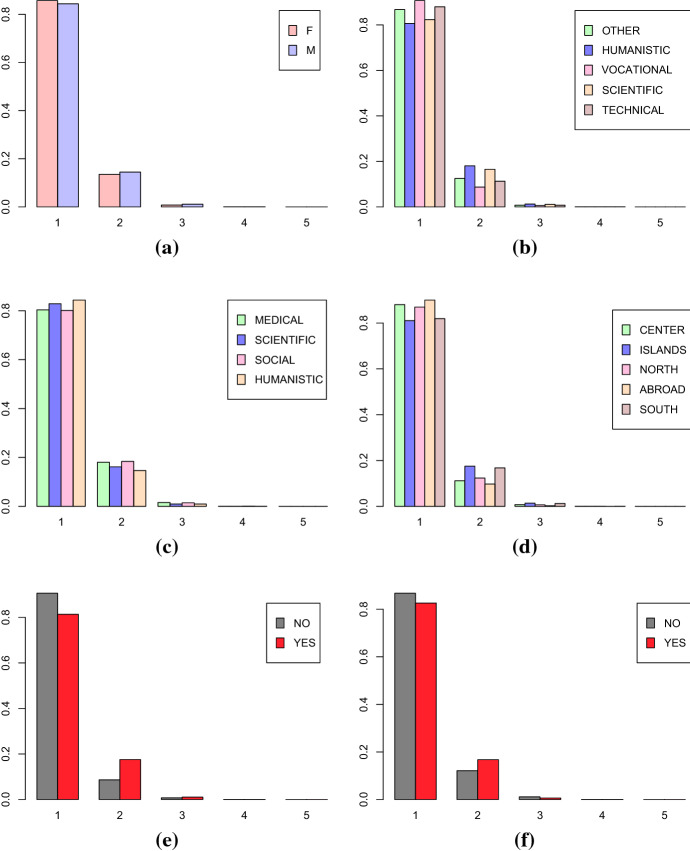
Distribution of the number of loops in the graphs representing the mobility trajectories of every students conditioned to **a** gender, **b** high school, **c** disciplinary area, **d** geographical area, **e** Bachelor’s Degree, **f** Master’s Degree

**Fig. 11:**
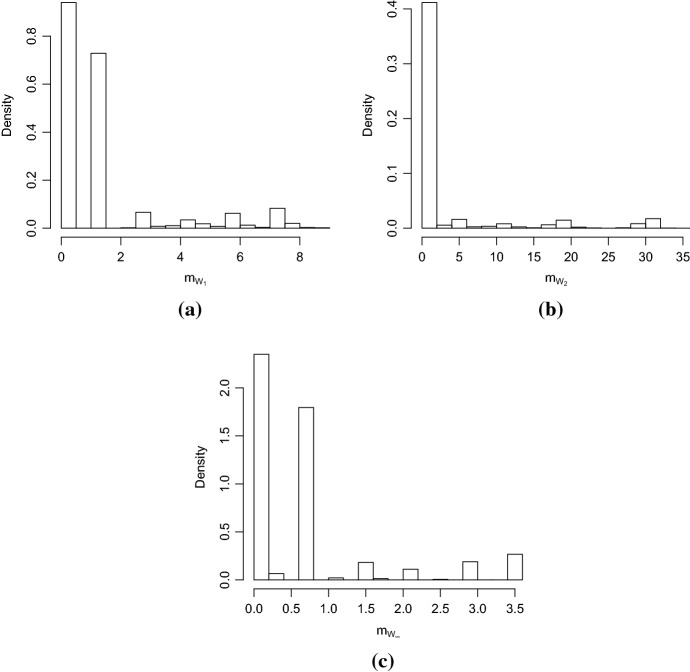
Distribution of the mobility index *m* computed for the persistence diagrams of the graphs of every students using **a** Wasserstein distance $p=1$, **b** Wasserstein distance $p=2$, **c** bottleneck distance $p=\infty$

**Fig. 12:**
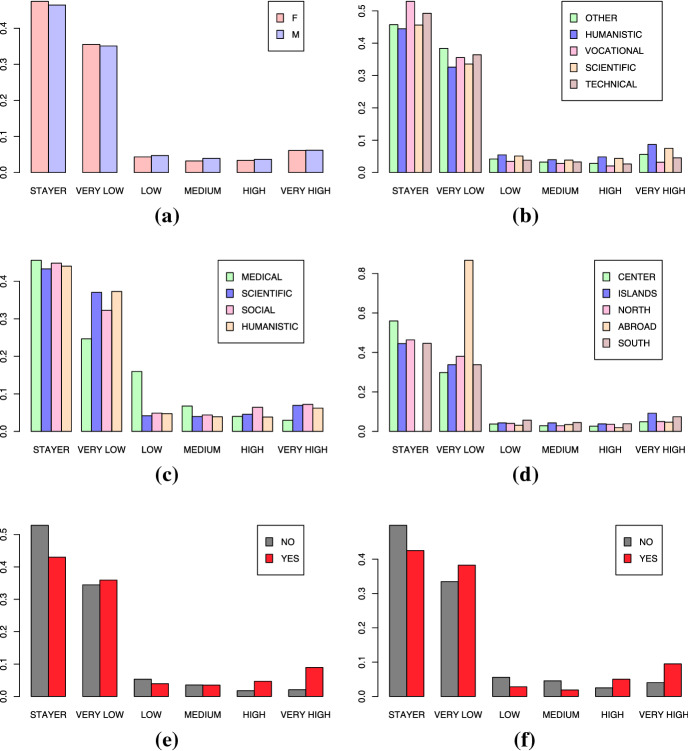
Distribution of the categorized mobility index *m* ($p=1$) conditioned to **a** gender, **b** high school diploma, **c** disciplinary area, **d** geographical area, **e** Bachelor’s Degree, **f** Master’s Degree

We are interested in students’ migratory flows, i.e. the mobility flows that occur after the high school diploma, over the Italian territory, from region to region. As in most of the related literature (see Attanasio and Priulla (2020) and the reference therein), the twenty Italian regions are grouped into four macro-regions: North, Center, South and Islands. Due to low numerical consistency, 2928 international students, 1.2% of the 2008 cohort, are excluded from the following analysis. Table 3 displays the distributions of the 240560 Italian students enrolled in an Italian university in 2008, by the macro-region of residence (in row) the macro-region of enrolment (in column), gender (A), and final high school mark (B) (defined as higher, if it is higher than 89/100, lower otherwise). Students are predominantly females (56.2%); 39.8% of students are resident in the North of Italy, 20% in the Center, 28.7% in the South and 11.5% in the Islands. A first indication of the entity of the Italian high school mobility phenomenon can be given looking at the distribution of the Italian students per macro-region of residence before and at the year of enrolment (2008). The larger high school mobility flows are from the South towards the Center (9.98%) and the North (7.15%), followed by those from the Islands towards the North (8.18%) and the Center (4.95%). The high school mobility flows of the North and the Center are low, roughly under 2% and 10%, respectively. This result is in line with the analysis carried out by Attanasio and Priulla (2020), in which the authors identify unidirectional mobility flows from “Mezzogiorno” (South and Islands) to the Center-North of Italy. In both the South and the Islands, the high school mobility flows of males students are proportionally wider than those of females students, and those of students with a higher final mark are wider than those with a lower mark. These findings are in agreement with what stated in Attanasio and Priulla (2020); D’Agostino et al. (2019), in which the authors notice: (i) gender difference in the entity of the mobility flow, but no difference in the macro-region in which they move and (ii) higher mobility for students with higher high school diploma. Figure 5 displays the distributions of the students from the South and the Islands by their macro-region of residence before and at the year of enrolment (2008) and the type of high school diploma. The flows from the North and the Center are omitted due to low numerosity. Using the definition provided in Attanasio and Enea (2019), we define as *high school movers* the students enrolled out of the region in which they obtained their high school diploma and *high school stayers* otherwise. The type of high school diploma seems to influence the mobility status of the students from the South-Islands part of the country: students with a humanistic or scientific high school diploma tend to move more (Fig. 5). This is in line with D’Agostino et al. (2019) that claim that movers are overrepresented among students who are enrolled in academic schools (“Liceo Scientifico” or “Liceo Classico”), while the majority of students who earned a diploma in vocational or technical schools tends to migrate less. In Figures 6, 7, 8, the mobility flows of the students that obtained their high school diploma in the macro-region South and Islands in 2008 (or in a year before 2008) are shown. The macro region of residence from the year of the diploma (Dipl.08 in Figures 6, 7, 8) to 2014 is shown and the resulting mobility flows are compared by gender, final mark, and type of high school diploma. For better visualisation exigencies the mobility flows from Northern and Central Italy are excluded. For the same reason we decided to consider the mobility flows only up to 2014 rather than 2016, given that no relevant flows’ changes are observed in this time period.

**Table 3:** Distribution of the Italian students by macro-region of residence, macro-region of enrolment, gender (A) and final high school mark (B)

		Macro-Region of enrolment										
Macro-region of residence	Gender	North	Center	South	Islands	Total	Final mark	North	Center	South	Islands	Total
North	Female	52757	897	133	60	53847	Lower	71660	1172	247	63	73142
		*97.98*	*1.67*	*0.25*	*0.11*	*56.2*		*97.97*	*1.60*	*0.34*	*0.09*	*78.9*
	Male	41101	730	159	24	42014	Upper	19123	397	39	19	19578
		*97.83*	*1.74*	*0.38*	*0.06*	*43.8*		*97.68*	*2.03*	*0.20*	*0.10*	*21.1*
	Total	93858	1627	292	84	95861	Total	90783	1569	286	82	92720
		*97.91*	*1.70*	*0.30*	*0.09*	*39.8*		*97.91*	*1.69*	*0.31*	*0.09*	*38.5*
Center	Female	1066	24771	781	16	26634	Lower	1297	32962	1055	23	35337
		*4.00*	*93.01*	*2.93*	*0.06*	*55.5*		*3.67*	*93.28*	*2.99*	*0.07*	*76.0*
	Male	991	19820	555	18	21384	Upper	598	10279	272	10	11159
		*4.63*	*92.69*	*2.60*	*0.08*	*44.5*		*5.36*	*92.11*	*2.44*	*0.09*	*24.0*
	Total	2057	44591	1336	34	48018	Total	1895	43241	1327	33	46496
		*4.28*	*92.86*	*2.78*	*0.07*	*20.0*		*4.08*	*93.00*	*2.85*	*0.07*	*19.3*
South	Female	2391	3588	31834	807	38620	Lower	3115	4672	40886	776	49449
		*6.19*	*9.29*	*82.43*	*2.09*	*56.0*		*6.30*	*9.45*	*82.68*	*1.57*	*72.3*
	Male	2538	3294	24112	392	30336	Upper	1678	2176	14707	409	18970
		*8.37*	*10.86*	*79.48*	*1.29*	*44.0*		*8.85*	*11.47*	*77.53*	*2.16*	*27.7*
	Total	4929	6882	55946	1199	68956	Total	4793	6848	55593	1185	68419
		*7.15*	*9.98*	*81.13*	*1.74*	*28.7*		*7.01*	*10.01*	*81.25*	*1.73*	*28.4*
Islands	Female	1030	678	120	13993	15821	Lower	1564	920	176	17764	20424
		*6.51*	*4.29*	*0.76*	*88.45*	*57.1*		*7.66*	*4.50*	*0.86*	*86.98*	*74.1*
	Male	1238	694	99	9873	11904	Upper	670	442	40	6002	7154
		*10.40*	*5.83*	*0.83*	*82.94*	*42.9*		*9.37*	*6.18*	*0.56*	*83.90*	*25.9*
	Total	2268	1372	219	23866	27725	Total	2234	1362	216	23766	27578
		*8.18*	*4.95*	*0.79*	*86.08*	*11.5*		*8.10*	*4.94*	*0.78*	*86.18*	*11.5*
Overall	Female	57244	29934	32868	14876	134922	Lower	77636	39726	42364	18626	178352
		*42.43*	*22.19*	*24.36*	*11.03*	*56.1*		*43.53*	*22.27*	*23.75*	*10.44*	*75.8*
	Male	45868	24538	24925	10307	105638	Upper	22069	13294	15058	6440	56861
		*43.42*	*23.23*	*23.59*	*9.76*	*43.9*		*38.81*	*23.38*	*26.48*	*11.33*	*24.2*
	Total	103112	54472	57793	25183	240560	Total	99705	53020	57422	25066	235213
		*42.86*	*22.64*	*24.02*	*10.47*	*100.0*		*42.39*	*22.54*	*24.41*	*10.66*	*97.8*

Percentage values in italics

From Figs. 6, 7 and 8 some results need to be remarked:

- Gender plays a role in the mobility flows of the students moving from the South and from the Islands: among the student that have obtained their diploma in the Italian Islands, 10.4% of males have enrolled in 2008 (Enrol.08) in a Northern university and 5.8% in a Central one, whereas for female students these percentages are respectively 6.5% and 4.3%; among the student that have obtained their diploma in South of Italy, 6.2% of males have enrolled in a Northern university, 9.3% in a Central one, whereas for female students these percentages are respectively 8.4% and 10.9% (Fig. 6).
- The final high school mark seems to influence the final destination of the students moving from the Islands: students with a higher final-mark have a higher propensity to move than those with a lower one, also in this case especially from the South (22.5% and 17.3%, respectively) and from the Islands (16.1% and 13%, respectively). At the time of enrolment, the largest flows of students with high and low marks are those from the South towards the Center (11.5% and 9.5%, respectively), followed by those towards the North (8.9% and 6.3%, respectively); the largest flows from the Islands are those towards the North, (9.4% and 7.7%, respectively), followed by those towards the Center (6.2% and 4.5%, respectively). In the subsequent years, the flows first experience a decrease and then an increase again in 2012 and 2013, mainly from the Islands and towards the North (Fig. 7).

- As regards the type of high school diploma, here just the mobility flows of the students that have obtained a humanistic or scientific high school diploma are represented because of their higher propensity to move: 10.1% and 9.4%, and 16% and 10% are the students with humanistic and scientific high school diploma moving from the Island and from the South (Fig. 8).
- Generally speaking mobility flows seem to decrease over time, but for the student that have obtained their diploma in the Italian Islands an increase from the fourth to the sixth year is observed, indicating some return flows.
- In 2012, although the number of students decreased compared to 2011, the number of movers increased, likely due to the enrolment in a Master’s degree course.

### Topological data analysis

For measuring the mobility dynamic of the Italian students, previously described in Sect. 4.1, the Topological Data Analysis (TDA) approach previously described is employed. The first step is creating the graphs for the mobility trajectories. To this purpose, the place of residence of the Italian students from the year of the diploma until 2017 is used. Figures 9 and 10 show the distributions of the number of vertices and the number of loops of the final graphs corresponding to the mobility trajectories of the Italian students conditioned to the levels of explanatory variables: gender (Female, Male) (a), high school (Humanistic, Scientific, Technical, Vocational, Other) (b), discipline area (Medical, Scientific, Social, Humanistic) (c), geographical area (North, Center, South, Islands, Abroad) (d), Bachelor’s degree (Yes, No) (e), Master’s degree (Yes, No) (f).

Afterwards, the mobility dynamic is evaluated making use of persistence homology. First, the persistence diagram for each graph representing the mobility trajectory is computed. Then the distance between every persistence diagram and the *stayer* persistence diagram is computed using Eq. 2. The resulting mobility indices computed using different metrics with equal weight for the number of vertices and for the number of loops are shown in Fig. 11.

Using the index based on the Wasserstein distance $p=1$, we describe the mobility using 6 levels: “Stayer”, “Very Low”, “Low”, “Medium”, “High”, “Very High”. With “Stayer”’ we mean that the difference with respect to the stayer persistence diagram is 0. The other levels are chosen picking as break points representative values of distance from the stayer persistence diagrams investigated in Table 2. In the “Very Low” class, we find students that moved immediately after their diploma. We classify them as having a very low mobility because they perform just one movement very early in their development process, building their path in another location. In the “Low” and “Medium” classes there are mainly the students that performed more than one change of residence, one of which is probably a return in an already visited location. In the “High” and “Very High” classes there are instead the students that performed more than one movement and that present uniquely an outgoing mobility. In Fig. 12, we describe the attitude of mobility conditioned to the levels of the explanatory variables previously mentioned using the categorised index. In Table 4 instead we report the summary values of the distribution of the index conditioned to the covariates level. The main findings are:

- With respect to the previous Sect. 4.1 in which the coomn classification of “stayer” and mover is used, and in which we found that gender was an influent factor for mobility, here there does not seem to be a very big difference in the mobility of male and female students. This is actually in line with the results reported in Columbu et al. (2021a), in which the authors consider a wider classification of stayer and mover defining five mobility profiles according to the time and the distance at which the students perform the moving. In fact, Columbu et al. (2021a) state that there are slight differences between the two groups of males and females in the probability of belonging to the five profiles.
- The students having a high school diploma in a scientific or humanistic area tend to have a higher mobility attitude with respect to the fellows that have a vocational or technical high school diploma. Students with a vocational diploma present the lowest mobility attitude as the median value of the mobility index is 0 (Table 4) ;
- The students that enrol in a degree course belonging to a Medical area tend to have a lower mobility attitude, implying a possible return on an already visited location. In most of the mobility studies, health disciplinary area is excluded, because of the peculiar national rules of admission to medical schools, alternatively as in Rizzi et al. (2021) the medical field of study is chosen as reference category of comparison because of its geographical homogeneity and its low propensity to mobility.;
- As previously observed, students which live in the South or in the Islands tend to have a higher mobility attitude especially with respect to the fellows from the Center and the North of the Italy. Students that are enrolled in the Center present the lowest mobility attitude (Median value = 0, Table 4). The students that come from abroad and enrol in an Italian university present mainly a “Very Low” mobility, because they mainly perform just one movement;
- Having a Bachelor’s or Master’s degree highly influences the mobility attitude of the students, confirming the conjecture that education plays a very important role in the mobility of the Italian students Iammarino and Marinelli (2015).

**Table 4:** Quantile (Q1, Q2, Q3), minimum, maximum, mean and standard deviation values of the mobility index *m* ($p=1$) conditioned to gender, high school diploma, disciplinary area, geographical area, bachelor’s degree, master’s degree

	Min	Q1	Q2	Q3	Max	Mean	SD
*Gender*							
F	0	0	1.275	1.275	8.782	1.311	1.959
M	0	0	1.275	1.275	8.839	1.366	1.992
*High school*							
Humanistic	0	0	1.275	1.275	8.782	1.600	2.224
Vocational	0	0	0	1.275	8.515	0.989	1.592
Scientific	0	0	1.275	1.275	8.782	1.488	2.123
Technical	0	0	1.275	1.275	8.839	1.162	1.785
Other	0	0	1.275	1.275	8.782	1.278	1.876
*Disciplinary area*							
Medical	0	0	1.275	2.550	8.631	1.395	1.798
Scientific	0	0	1.275	1.275	8.782	1.486	2.072
Social	0	0	1.275	1.275	8.839	1.580	2.185
Humanistic	0	0	1.275	1.275	8.782	1.403	1.985
*Macro-region*							
North	0	0	1.275	1.275	8.782	1.257	1.834
Center	0	0	0	1.275	8.782	1.084	1.858
South	0	0	1.275	1.275	8.782	1.501	2.105
Islands	0	0	1.275	1.275	8.839	1.584	2.225
Abroad	1.275	1.275	1.275	1.275	8.284	1.759	1.402
*Bachelor’s degree*							
Yes	0	0	1.275	1.275	8.782	1.597	2.219
No	0	0	0	1.275	8.834	0.953	1.466
*Master’s degree*							
Yes	0	0	1.275	1.275	8.782	1.597	2.247
No	0	0	1.275	1.275	8.839	1.595	1.757

## Discussion and conclusions

In this paper we showed an application of persistence homology for evaluating the mobility dynamic of the Italian students. The idea is to represent the mobility trajectories of the students with graphs made of vertices corresponding to the different locations visited by the students and directed edges representing the performed transitions and to distinguish the movements that students perform in terms of their importance. The new approach allows to encapsulate in a unique measure the most influent factors for mobility behaviours. More specifically, we take into account: (i) the number of the transitions that the student performs; (ii) when the transition has been performed and the time spent in a specific location: this means that moving immediately after the high school diploma is different than moving after the Bachelor’s or the Master’s degree. The idea is that the more time the student spends in a specific location, the more that location and its context affect his/her development and growth process, the more significant will that transition be in the definition of his/her mobility attitude; (iii) returns to an already visited location. Using zigzag persistence homology that allows to evaluate the dynamic nature of the graphs over time, a new mobility index based on the distance from the persistence diagram of the ideal stayer student is proposed.

After having explored the mobility structure of the Italian students that in 2008 enrolled in an Italian university using classical descriptive statistics, the new methodology is applied for the analysis of the Italian interregional mobility from the year of the diploma until 2017. The new mobility index, with respect to the classical definition of “stayer” and “mover”, is able to capture the mobility dynamic over time using the lifetime of vertices and loops and to give a quantitative measure of the attitude to mobility. The new methodology confirms what found in the descriptive section and in the related literature, conveying the information in a compact and accurate way. The main findings are that graduates and students coming from the South or from the Islands have a higher mobility attitude with respect to their fellows that did not achieve the title and to the students coming from the Central regions. Gender slightly influences the mobility behaviour. The relation between the mobility index and the discipline area of the diploma and of the degree course in which the students enrol suggests that students with a scientific or humanistic diploma and students that enrol in scientific, humanistic or social area tend to have a higher mobility attitude with respect to their fellow with a vocational or technical diploma that enrol in medical area.

The low mobility attitude of medical students is an additional confirm of the goodness of the method that captures the mobility dynamic of medical students whose mobility is conditioned to the national rules for university enrolment and specialisation. However, some more general considerations are needed. The individual graphs of the students are created using the province of residence declared by the students every year. This choice probably prevents us to know about unregistered movements and commuting students. At the same time, this reduce the entity of the second problem, that is that we do not give weights to the graph edges. Not giving weight to the graph edges means giving the same weight to the transitions that occur within and between regions. Considering the province of residence and hence just official movement we assume that mostly significative transitions occurring between regions will be recorded. A possible solution is to define a system of weight for the graph edges using the physical distance associated to the transition (though this might penalise students that live in poorly connected places) or a different weight according to within and in between regions transitions. Developments in this direction will be made. Another extension regards the possibility of applying persistence homology directly on graphs (Bergomi and Vertechi 2020), including other important graph specific features such as cliques or triangles that will allow the study of the persistence of specific students’ mobility patterns. Lastly, the mobility structure of the Italian students, but in general of the European students, will be highly affected after Covid 19 pandemics. In fact, the restrictive measures and the “lockdown” adopted by the governments for reducing the virus spread, influence the mobility trajectories, liming the movements. Moreover, the economic crisis caused by the pandemics might be stronger than 2007 crisis. The entity of the mobility phenomenon will change but also its nature. We might observe *virtual mobility*. We therefore aim to repeat the analysis for understanding the mobility nature and dynamic after the pandemics and to compare it with the one studied in this paper.

## Appendix

See Table 5

**Table 5:** Code list of the Italian provinces

Code	Province	Region	Code	Province	Region
AG	Agrigento	Sicilia	MO	Modena	Emilia Romagna
AL	Alessandria	Piemonte	MS	Massa-Carrara	Toscana
AN	Ancona	Marche	MT	Matera	Basilicata
AO	Aosta	Valle d’Aosta	NP	Napoli	Campania
AP	Ascoli-Piceno	Marche	NO	Novara	Piemonte
AQ	L’Aquila	Abruzzo	NU	Nuoro	Sardegna
AR	Arezzo	Toscana	OG	Ogliastra	Sardegna
AT	Asti	Piemonte	OR	Oristano	Sardegna
AV	Avellino	Campania	OT	Olbia Tempio	Sardegna
BA	Bari	Puglia	PA	Palermo	Sicilia
BG	Bergamo	Lombardia	PC	Piacenza	Emilia Romagna
BI	Biella	Piemonte	PD	Padova	Veneto
BL	Belluno	Veneto	PE	Pescara	Abruzzo
BN	Benevento	Campania	PG	Perugia	Umbria
BO	Bologna	Emilia Romagna	PI	Pisa	Toscana
BR	Brindisi	Puglia	PN	Pordenone	Friuli Venezia Giulia
BS	Brescia	Lombardia	PO	Prato	Toscana
BT	Barletta-Andria-Trani	Puglia	PR	Parma	Emilia Romagna
BZ	Bolzano	Trentino Alto Adige	PT	Pistoia	Toscana
CA	Cagliari	Sardegna	PU	Pesaro-Urbino	Marche
CB	Campobasso	Molise	PV	Pavia	Lombardia
CE	Caserta	Campania	PZ	Potenza	Basilicata
CH	Chieti	Abruzzo	RA	Ravenna	Emilia Romagna
CI	Carbonia Iglesias	Sardegna	RC	Reggio-Calabria	Calabria
CL	Caltanissetta	Sicilia	RE	Reggio-Emilia	Emilia Romagna
CN	Cuneo	Piemonte	RG	Ragusa	Sicilia
CO	Como	Lombardia	RI	Rieti	Lazio
CR	Cremona	Lombardia	RN	Rimini	Emilia Romagna
CS	Cosenza	Calabria	RO	Rovigo	Veneto
CT	Catania	Sicilia	RM	Roma	Lazio
CZ	Catanzaro	Calabria	SA	Salerno	Campania
EN	Enna	Sicilia	SI	Siena	Toscana
FC	Forli-Cesena	Emilia Romagna	SO	Sondrio	Lombardia
FE	Ferrara	Emilia Romagna	SP	La-Spezia	Liguria
FG	Foggia	Puglia	SR	Siracusa	Sicilia
FI	Firenze	Toscana	SS	Sassari	Sardegna
FM	Fermo	Marche	SV	Savona	Liguria
FR	Frosinone	Lazio	TA	Taranto	Puglia
GE	Genova	Liguria	TE	Teramo	Abruzzo
GO	Gorizia	Friuli Venezia Giulia	TN	Trento	Trentino Alto Adige
GR	Grosseto	Toscana	TO	Torino	Piemonte
IM	Imperia	Liguria	TP	Trapani	Sicilia
IS	Isernia	Molise	TR	Terni	Umbria
KR	Crotone	Calabria	TS	Trieste	Friuli Venezia Giulia
LC	Lecco	Lombardia	TV	Treviso	Veneto
LE	Lecce	Puglia	UD	Udine	Friuli Venezia Giulia
LI	Livorno	Toscana	VA	Varese	Lombardia
LO	Lodi	Lombardia	VB	Verbania	Piemonte
LT	Latina	Lazio	VC	Vercelli	Piemonte
LU	Lucca	Toscana	VE	Venezia	Veneto
MB	Monza-Brianza	Lombardia	VI	Vicenza	Veneto
MC	Macerata	Marche	VR	Verona	Veneto
ME	Messina	Sicilia	VS	Medio Campidano	Sardegna
MI	Milano	Lombardia	VT	Viterbo	Lazio
MN	Mantova	Lombardia	VV	Vibo-Valentia	Calabria
